# The Training of Nurses

**Published:** 1924-07

**Authors:** Wilmot Herrringham


### THE TRAINING OF NURSES.

### BY SIR WILMOT HERRRINGHAM, K.C.M.G., M.D., ETC.

We were in the habit, Sir Wilmot began, of thinking that the training of nurses was better in England than in any other country. That might or might not be so, but at any rate such training was not of very long standing. As recently as 1843 "Martin Chuzzlewit" appeared, and there burst upon the scene that eponymous heroine of the nursing world, Sarah Gamp. She was no great caricature of nursing in her time, and her friend Betsy Prig was connected with his own hospital—St. Bartholomew's. In 1850, when Florence Nightingale wanted to get her training, she had to go abroad because she could not get it in England. As far as he knew, the first serious training school of nursing in England was that set up at St. Thomas's hospital in 1860 by national subscription in commemoration of Florence Nightingale's work in the Crimea.

### The Beginning of Training.

Sir Wilmot proceeded to review the various stages through which the training of nurses had passed, from 1876 onwards. In that year the authorities found there was some diminution in the supply, and that was ascribed to the menial work then required of nurses. It was the duty of ward-maids to scrub and clean the wards, to fetch and carry, and cook such simple things as were required. Evidently up to that time the nurses had to do that. In 1877—the year in which he himself first joined the hospital as a medical student—the first real regulations were introduced for training. The period of training was fixed at one year; the age of admission at between 20 and 35; an examination was instituted and a certificate given. In 1880 the period of training was extended to two years, and in 1882 further extended to three years, and the entrance age raised to 23. With the extension of the period of training to three years an examination was set at the end of the first year as well as that at the end of the time.

### Development.

In 1890 there were considerable changes. The hospital adopted the system of making the nurses contract to the institution for a period of years. They required a nurse to undergo certain preliminary examinations as to health, education and general intelligence, and in certain special knowledge. She had also to be able to explain the symbols and signs used in prescriptions. All this foreshadowed what was now very much talked about and was actually in existence at the London Hospital—preliminary schools of training. That system lasted until 1914, when the age was lowered to 22, and in 1918 again lowered to 21. In 1919 a committee reported, and nurses were required to undergo a course of lectures. All this was of peculiar interest to him, because he remembered very well the old style of sister—very capable, very practical, and some of them very kind and compassionate, but quite unfitted to do the work now required of the modern nurse, who had developed to suit the needs of the modern doctor. She was not more different from the old sisters of the wards than the modern doctor was from the men who first taught him.

### Where are the Educated Ladies?

But he thought there was one aspect in which nursing to-day was rather worse off than when the development began. When he was young a great many educated ladies were taking up the profession, partly, he supposed, owing to the enthusiasm created by Miss Nightingale, but chiefly to the fact that nursing was one of the few means of earning a living which educated women could then pursue. Nowadays a woman could be a barrister, a doctor, a minister of religion, or even a member of Parliament. It was, therefore, not surprising that they did not come in crowds into a poorly paid and laborious profession such as nursing, and he was quite sure that nursing was poorer for their absence.

### The General Nursing Council.

Sir Wilmot proceeded to describe the developments since the Registration of Nurses Act of 1919 had thrown the education of nurses on to the shoulders of the General Nursing Council, of which he was chairman. They had to consider what policy to adopt. It was quite well known that the sort of training he had described was given in a great many schools, but there were also a great many other schools—probably a majority—in which the training was very much more backward. The Council had to consider whether it would indulge the more backward schools and set a standard lower than the best, or whether it should keep to the best standard. The Council took the view that if it took the lower standard it would, first, be justly blamed by the public for whose security the Council was set up; it would, secondly, bring the Register into contempt, and probably all the best trained nurses would refuse to register, and, lastly, it would lower the status and prestige of the nursing profession itself. The Council decided, therefore, to adopt the standard of the best schools, and to hope that the more backward ones would raise their training to the higher level. The Council drew up a syllabus of training which in its essentials was based on the methods in use at the most forward schools, and submitted it to the Minister for sanction. The result would have been that the training would have been made compulsory on all schools, but immediately a great outcry was raised, especially by Poor Law authorities, many of whom said that their schools could not possibly teach the subjects laid down. The Minister yielded to the outcry and refused to sign the syllabus. That was most fortunate. If the scheme had been sanctioned it would have become compulsory by law and unchangeable, and, since the very last thing that anybody who knew anything about education wanted was to stereotype a method, he thought it was most fortunate that the Minister refused to sanction it. But however much they might dislike stereotyped methods, certain things had to be fixed, and among them were the subjects in which the candidate was going to be examined.

